# Diagnostic accuracy of active pulmonary tuberculosis screening during detention admission: a systematic review

**DOI:** 10.25122/jml-2024-0155

**Published:** 2024-07

**Authors:** Stephanie Pape, Kabiru Gulma, Siddharudha Shivalli, Laurent Cleenewerck de Kiev

**Affiliations:** 1 Department of Global Health, Euclid University, Bangui, Central African Republic; 2 Center for Evaluation, London School of Hygiene & Tropical Medicine, London, United Kingdom

**Keywords:** early diagnosis, mass screening, prisons, sensitivity and specificity, tuberculosis, 95% CI, 95% Confidence Intervals, AUC, Area Under the Curve, BCG, Bacille Calmette-Guérin, CXR, Chest Radiography, DOTS, Directly Observed Therapy Shortcourse, ECDC, European Centre for Disease Prevention and Control, EPTB, Extrapulmonary Tuberculosis, FL LPA, First-Line Line Probe Assay, FN, False Negative, FP, False Positive, H0, Null Hypothesis, H1, Alternative Hypothesis, HBC, High-Burden Country, HIV, Human Immunodeficiency Virus, IGRA, Interferon-Gamma Release Assay, LAM, Lipoarabinomannan, LMICs, Low- and Middle-Income Countries, LPA, Line Probe Assay, LTBI, Latent Tuberculosis Infection, MDR TB, Multidrug-Resistant Tuberculosis, NAAT, Nucleic Acid Amplification Test, NMA, Network Meta-Analysis, PICOS, Population, Intervention, Comparison, Outcomes, Setting, PRISMA, Preferred Reporting Items for Systematic Reviews and Meta-Analyses, PROSPERO, International Prospective Register of Systematic Reviews, PTB, Pulmonary Tuberculosis, QUADAS-2, Quality Assessment of Diagnostic Accuracy Studies (Revised Tool), ROC, Receiver Operating Characteristic, TB, Tuberculosis, TB-LAMP, Loop-Mediated Isothermal Amplification, TN, True Negative, TP, True Positive, TST, Tuberculosis Skin Test (Mendel-Mantoux Test), WHO, World Health Organization

## Abstract

Individuals entering incarceration are at high risk for infectious diseases, other ill conditions, and risky behavior. Typically, the status of active pulmonary tuberculosis (PTB) is not known at the time of admission. Early detection and treatment are essential for effective TB control. So far, no study has compared the diagnostic accuracy of various TB screening tools in detention using a network meta-analysis (NMA). We aimed to investigate the diagnostic accuracy of active PTB screening tests upon detention admission. We searched PubMed, Global Index Medicus, the Cochrane Library electronic databases, and grey literature for publications reporting detention TB entry screening in March 2022 and January 2024. Inclusion was non-restrictive regarding time, language, location, reference standards, or screening tests. Eligible study designs comprised comparative, observational, and diagnostic studies. Publications had to report TB screening of individuals entering confinement and provide data for diagnostic accuracy calculations. The QUADAS-2 tool was designed to assess the quality of primary diagnostic accuracy studies. This systematic review was registered with PROSPERO (CRD42022307863) and conducted without external funding. We screened a total of 2,455 records. Despite extensive searching, no studies met our inclusion criteria. However, we identified evidence revealing key differences in screening algorithm application. In conclusion, more diagnostic accuracy data on TB screening algorithms for detention admission worldwide needs to be collected. We recommend that global TB initiatives set up multi-site studies to investigate the diagnostic accuracy of TB screening on admission in low- and high-prevalence criminal justice systems. Further network meta-analyses of these studies could inform policymakers and public health experts to establish or fine-tune TB control in detention settings.

## INTRODUCTION

Despite successfully reducing tuberculosis (TB) mortality, the disease remains a leading cause of death worldwide [1]. Incarcerated persons belong to the high-risk groups for TB infection and illness. That is due to behavioral risk factors (e.g., alcohol and substance use disorders), comorbidities such as human immunodeficiency virus (HIV) infection or hepatitis, and the confinement environment favoring air-borne transmissions [2-4].

**Figure 1:**
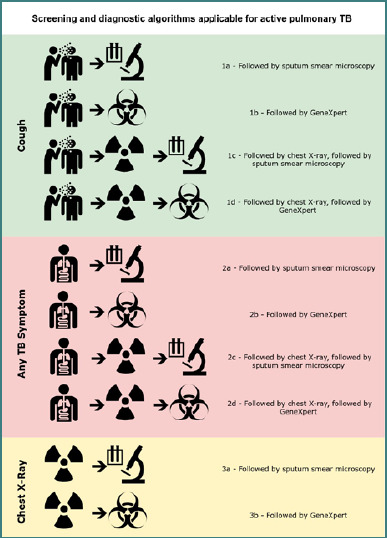
Screening and diagnostic algorithms applicable for active PTB [7]

The criminal justice authorities are commonly responsible for healthcare in detention [5]. The revised European Prison Rules recommend establishing a standard medical TB entry screening encompassing other ill conditions for every newly arrived individual. According to the World Health Organization (WHO), TB control measures in detention should comprise active and passive case-finding based on three strategies: self-referral, screening at entry, and active case-finding in residents [5,6]. Generally, WHO experts recommend at least one screening test supplemented by one diagnostic test for systematic TB screening [7]. The guidelines provide ten algorithms combining established screening and diagnostic tests for a predefined target population (Figure 1). The algorithms differ by the screening approach, i.e., cough, TB symptoms, or chest X-ray (CXR) screening, followed by a specific diagnostic test to confirm active pulmonary TB (PTB). Accepted confirmatory tests are sputum examinations, including smear microscopy, WHO-approved nucleic acid amplification tests (NAAT), e.g., GeneXpert MTB/RIF, and bacterial culture of *M. tuberculosis* [5,8]. Health services in detention settings may use these tests as reference standards, with bacterial culture as the gold standard of TB detection [8,9].

Optimally, every newly admitted person participates in TB screening and is kept separate from peers and the general facility personnel until considered unlikely to have active PTB [5,6]. Those who test positive on the initial screening test, including true positives (TP) and false positives (FP), will undergo further confirmatory testing. While true positives will receive appropriate treatment, diagnostically unconfirmed false positives might be subjected to unnecessary empire-driven drug therapy, a concerning issue in light of the global rise in multidrug-resistant TB (MDR TB). Conversely, individuals who test negative on the initial screening, whether true negatives (TN) or false negatives (FN), typically do not proceed to additional TB testing. This approach poses a significant risk of TB transmission within detention facilities, as false negatives may unknowingly spread the disease to peers, personnel, and visitors. That is particularly true for asymptomatic individuals or those not reporting symptoms voluntarily as needed for passive case-finding [8].

Several studies have investigated the diagnostic accuracy of various TB screening tests and algorithms within custodial populations [2,3,6,10–14]. However, exploratory PubMed literature searches on July 3, 2020, and February 18, 2021, indicated that those publications exclusively report direct comparisons of a limited number of TB screening tests or algorithms (results not shown). Furthermore, cost-effectiveness analyses of TB detection in detention settings typically utilize TB prevalence data, transmission models, and diagnostic strategies. However, they often overlook the diagnostic accuracy and practical applicability of screening tools in low-resource environments, such as those found in low- and middle-income countries (LMICs) [15–17].

### Diagnostic accuracy definition

Diagnostic test methods are intended to diagnose or monitor disease states and must meet regulatory standards for quality, efficacy, and safety, similar to medicinal products. These standards need to be established and validated within clinical development programs [18]. Demonstrating diagnostic efficacy involves assessing diagnostic accuracy, defined by parameters such as sensitivity, specificity, predictive values, and receiver operating characteristic (ROC) curves [19]. A single reference standard should be consistently used across the entire study population for test accuracy analysis. Sensitivity and specificity reflect the overall diagnostic accuracy of a test and are specific parameters independent of disease prevalence [20]. These metrics are frequently utilized, as highlighted in this systematic review. Parallel interpretation of both variables is always recommended when evaluating diagnostic accuracy [19]. We hypothesized that findings from a systematic review and network meta-analysis (NMA) about the diagnostic accuracy of TB entry screening in detention facilities would identify a screening algorithm that meets pre-defined sensitivity and specificity thresholds and is superior in balanced diagnostic accuracy.

1. Meeting pre-defined thresholds - binary tests
  According to a WHO consensus meeting in April 2014, a TB screening test should achieve at least 90% sensitivity and 70% specificity [21]. These two values of vital diagnostic accuracy measures would set up the 'minimally acceptable criteria’ (MAC) for a pre-defined test performance threshold that screening algorithms had to meet to be beneficial for screening purposes. Therefore, the statistical hypotheses for binary parameters are as follows [22]:
  H_0_: {Sensitivity_Index_ < 90% and/or Specificity_Index_ < 70%} and
  H_1_: {Sensitivity_Index_ ≥ 90% and Specificity_Index_ ≥ 70%}
2. Meeting the target region - continuous tests
  For continuous tests evaluated through ROC curves, the pre-defined sensitivity and specificity values form a 'target region’ in the upper left corner of an ROC diagram. A screening test with an ROC that did not cross the target region would be unacceptable. Thus, the statistical hypotheses are [22]:
  H_0_: ROC (0.30) ≤ 0.90 and
  H_1_: ROC (0.30) > 0.90
3. Balanced diagnostic accuracy – binary tests
  Only screening algorithms with screening test components with pre-defined thresholds of 90% sensitivity and 70% specificity are considered sufficient for TB screening activities and, thus, transferred to balanced diagnostic accuracy (BDA) estimation. BDA is calculated as the average of sensitivity and specificity [23]. For TB screening tests that meet these criteria, the BDA should be at least {(0.90 + 0.70)/2} = 0.80. We expected that screening tests meeting the MAC would differ in BDA, with one index test reaching the highest value corresponding to superiority. Thus, the statistical hypotheses were:
  H_0_: BDA _Test 1_ = BDA _Test 2B_ =…= BDA _Test n_ and
  H_1_: Not all BDA _Test i_ are equal (i = 1, 2,.., n)

We aimed to conduct a systematic review and NMA to determine the most accurate algorithm for detecting active PTB in individuals upon admission, i.e., the combination of screening and diagnostic tests, while also considering service feasibility in resource-limited criminal justice systems within high-burden TB countries.

## MATERIAL AND METHODS

### Search strategy and selection criteria

One author (SP) performed literature searches in PubMed, Global Index Medicus, the Cochrane Library, and grey literature sources from March 6 to 13, 2022, merged the search results, and removed duplicates. A search update in the bibliographic databases occurred on January 16, 2024. The search strategy is presented in Appendix 1. Table 1 provides an overview of the key characteristics addressed in the review using the PICOS algorithm [24].

**Table 1:** Screening for active PTB at prison entry – PICOS algorithm [24]

Screening for active PTB at prison entry	
P	Newly arriving inmates of any age at entry in prison settings.
I	Active pulmonary TB case-finding by a screening algorithm.
C	A composite reference standard comprising bacteriological confirmation by solid/liquid culture, and/or positive sputum smear(s), and/or a WHO-endorsed nucleic acid amplification test (NAAT), e.g., GeneXpert MTB/RIF.
O	Diagnostic accuracy data, such as sensitivity, specificity, true positive, false-positive, true negative, and false-negative values.
S	Prisons, jails, and other custodial settings with the functioning as a prison (excluding migrant centers and police detention rooms).

Appendix 1

Regarding inclusion in our systematic review, we refrained from any restrictions concerning time, language, regions or countries where studies were conducted, or the reference standards and screening tests used. Given the expected limited number of publications dealing with the review topic, we initially accepted various study types for inclusion if the study’s objective and data provided aligned with our aim (Table 2). Eligible study designs included randomized controlled trials, non-randomized, prospective comparative studies, and observational studies (such as cohort, case-control, cross-sectional studies), and diagnostic accuracy studies. To qualify for inclusion, publications needed to focus on TB screening at the point of entry into detention facilities. We accepted study populations comprising newly admitted individuals in prisons, jails, and other settings that function as prisons, or detained individuals, including those in remand. Moreover, the studies had to provide quantitative data for calculating diagnostic accuracy.

**Table 2:** Inclusion and exclusion criteria [24]

	Inclusion	Exclusion
Study design	Randomized controlled trials (RCTs) Non-randomized, prospective comparative studies Observational studies (e.g., cohort studies, case-control studies) Cross-sectional studies Diagnostic studies	Narrative reviews Case reports/ case series Non-pertinent publication types (e.g., expert opinions, letters to the editor, editorials, comments, conference abstract/poster, news, consensus documents, chapter) Animal studies Genetic studies, biochemistry, or molecular studies Modeling studies Outbreak studies
Study characteristics	Study duration (not limited) Number of subjects (not limited)	Concerns about methodological quality (inherent methodology or insufficient methodology information)
Study population	Individuals in prisons, jails, and other settings that function as a prison Detained persons, including persons in remand	Persons in police custody Persons in migrant detention centers Facility personnel Any kind of visitors
Outcomes	Quantitative data applicable for diagnostic accuracy calculations	Lacking data applicable for diagnostic accuracy calculations

Two authors (KG and SP) independently screened all records by titles and abstracts. SP and AV conducted the full-text and updated screening, again blinded against each other. We performed all deduplication and screening procedures with the online tool Rayyan [25]. Excluded full-text publications were listed according to the criteria stated in Table 3 (data not shown). A third reviewer was available to resolve any discrepancies.

**Table 3:** Exclusion criteria for studies in the systematic review

Exclusion criteria	
E-1	Duplicate
E-2	Incorrect setting
E-3	No detention entry screening
E-4	Not screened for active PTB
E-5	Study design (e.g., comment, letter, editorial, narrative review, case report, case series)
E-6	Insufficient information about the study methodology
E-7	Insufficient information about screening procedure (tests used, simultaneous/ sequential testing, etc.)
E-8	Insufficient information about or different study population
E-9	No data about diagnostic accuracy measures (sensitivity, specificity, true positives, false positives, true negatives, false negatives)

### Data extraction and quality assessment

To ensure reliability in our systematic review, KG and SP initially piloted the Quality Assessment of Diagnostic Accuracy Studies-2 (QUADAS-2) tool on two randomly selected publications [26–28]. Satisfactory agreement occurred in all domains regarding the individual signal questions (data not shown). Discrepancies concerning index test and reference standard blinding and reproducibility did not lead to signal question changes. Following this preliminary assessment, both reviewers independently extracted data according to the review protocol and assessed the risk of bias with the QUADAS-2 tool, remaining blinded to each other’s assessments [26,29].

### Data analysis

We considered JASP 0.16.3-Debug and RStudio Cloud version 4.2.1 for descriptive statistics, individual, sensitivity, and network meta-analyses [30,31]. Visualization of the QUADAS-2 results was facilitated using the robvis web application [32].

### Systematic review

To evaluate the QUADAS-2 agreement between the two independent investigators, we used Krippendorff’s alpha as our primary measure [33]. Our study protocol was registered with the International Prospective Register of Systematic Reviews (PROSPERO) on March 2, 2022 (CRD42022307863) [34]. Further methodological details were published elsewhere [29].

## RESULTS

### Study selection

The literature searches identified 2,455 records: 1,151 from electronic databases and 1,304 from grey literature. After deleting 256 duplicates, KG and SP excluded 1,876 of 2,199 potentially eligible records. An additional 68 records were excluded during an updated search by AV and SP based on independent title and abstract screening. SP also identified six additional records through reference screening. AV and SP did a blinded assessment of the resulting 239 records eligible for a full-text review and 22 full texts from the update.

Per protocol, studies had to deliver data that allowed 2x2 contingency table calculations for an index test compared with the reference standard. Although several publications presented some valuable data, they lacked essential details or mixed entry screening information with mass or exit screening results. Therefore, the availability of information needed to estimate diagnostic accuracy parameters on penitentiary admission was insufficient.

**Figure 2:**
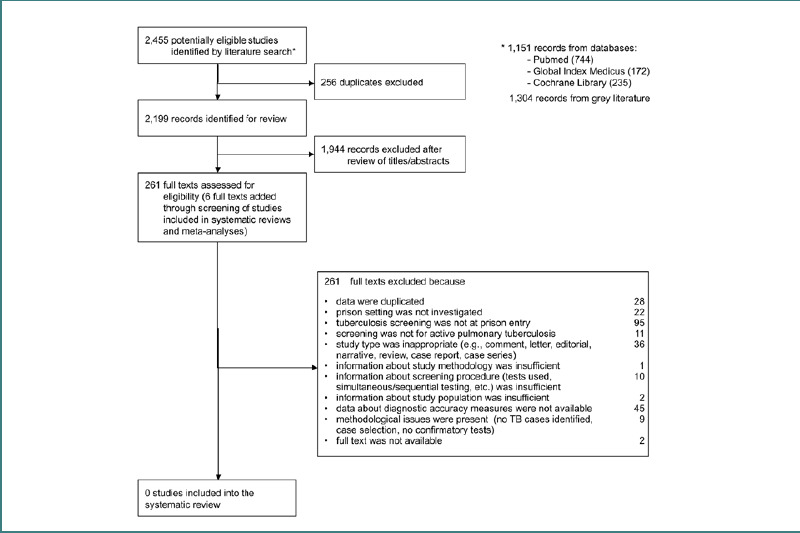
Study selection process (flow chart according to the PRISMA Statement) [35]

Consequently, none of the studies met our criteria to be included in our systematic review (Figure 2) [35]. Nevertheless, we identified 39 publications that reported TB screening on admission [27,28,36–72]. These revealed key differences in screening algorithm application. Apart from our methodology, we want to present and discuss this evidence narratively in the following section.

### Study characteristics

The main study characteristics of the publications reporting TB entry screening measures are listed in Table 4. The studies spanned from 1954 to 2022, with study durations ranging from three to 216 months. The majority of investigations regarding TB screening upon admission to detention facilities were conducted in the United States (*n* = 12). Of the countries investigated, ten were listed on the WHO TB high-burden country (HBC) list, nine on the multidrug-resistant TB (MDR-TB) list, and twelve on the TB/HIV HBC list [74]. Usually, low to upper-middle countries were ranked on one or more lists. Countries located on all three HBC lists were China, Ethiopia, India, and Zimbabwe. Among the 39 publications, 15 employed a cross-sectional study design, making it the most common study design. None of the studies were diagnostic accuracy studies. Eighteen (46%) studies were conducted in prisons, followed by 12 (31%) in jails. Nine (23%) publications researched TB entry screening system-widely.

**Table 4:** Main study characteristics

Author	Year	Country	Income	TB	MDR	HIV	Study type	Setting	Definition active PTB
Abascal [36]	2020	Peru	Upper-middle	N	Y	N	Retrospective case review	Prison	NR
Abeles [37]	1970	USA	High	N	N	N	Retrospective descriptive	System	NR
Al-Darraji [68]	2022	Malaysia	Upper-middle	N	N	N	Cross-sectional	Prison	Bacteriologically confirmed
Anderson [38]	1986	USA	High	N	N	N	Retrospective descriptive	System	NR
Askarian [39]	2001	Iran	Lower-middle	N	N	N	Prospective descriptive	Jail	Bacteriologically confirmed
Baird [72]	2022	South Africa	Upper-middle	Y	Y	Y	Prospective descriptive	Prison	Bacteriologically confirmed
Banu [40]	2015	Bangladesh	Low	N	Y	N	Prospective cohort	Jail	Bacteriologically confirmed^2^
Bellin [41]	1993	USA	High	N	N	N	Cross-sectional	Jail	Infectious PTB^5^
Bock [43]	1998	USA	High	N	N	N	Retrospective case review	System	NR
Bossard [42]	2020	Malawi	Low	N	N	Y	Prospective cohort	Prison	Bacteriologically confirmed^1^
Chevallay [44]	1983	Switzerland	High	N	N	N	Prospective cohort	Prison	Bacteriologically confirmed^1,4^
de Vries [45]	2020	Netherlands	High	N	N	N	Retrospective case review	System	NR
Degner [46]	2016	USA	High	N	N	N	Retrospective cohort	Jail	Bacteriologically confirmed^2^
Evrevin [47]	2021	France	High	N	N	N	Interventional/ Point-of-care	Prison	Bacteriologically confirmed^1^
Henostroza [48]	2013	Zambia	Low	Y	N	Y	Cross-sectional	Prison	Bacteriologically confirmed^1^
Kanyerere [49]	2012	Malawi	Low	N	N	Y	Retrospective descriptive	System	NR
Layton [50]	1997	USA	High	N	N	N	Cross-sectional	Jail	Bacteriologically confirmed^1^
Maggard [51]	2015	Zambia	Low	Y	N	Y	Cross-sectional	Prison	Bacteriologically confirmed^1^
Mandizvidza [52]	2020	Zimbabwe	Lower-middle	Y	Y	Y	Retrospective cohort	Prison	Bacteriologically confirmed^1^
Martin [53]	1994	Spain	High	N	N	N	Cross-sectional	Prison	Bacteriologically confirmed
Martin [54]	2001	Spain	High	N	N	N	Cross-sectional	Prison	Bacteriologically confirmed^1,3^
Meyers [55]	1956	USA	High	N	N	N	Retrospective descriptive	Jail	NR
Pelissari [56]	2018	Brazil	Upper-middle	Y	N	Y	Cross-sectional	Jail	Bacteriologically confirmed
Prasad [57]	2017	India	Lower-middle	Y	Y	Y	Cross-sectional	System	NR
Puisis [27]	1996	USA	High	N	N	N	Retrospective descriptive	Jail	Bacteriologically confirmed^1,2^
Reichard [58]	2003	USA	High	N	N	N	Retrospective descriptive	Jail	NR
Ritter [59]	2012	Switzerland	High	N	N	N	Retrospective descriptive	Jail	NR
Rudoi [60]	1990	Russian Federation	Upper-middle	Y	Y	N	Retrospective cohort	System	NR
Rutz [61]	2008	USA	High	N	N	N	Retrospective descriptive	Jail	NR
Sanchez [62]	2009	Brazil	Upper-middle	Y	N	Y	Prospective cohort	Prison	Bacteriologically confirmed^1^
Sanchez [28]	2013	Brazil	Upper-middle	Y	N	Y	Prospective cohort	Prison	Bacteriologically confirmed^1^
Saunders [63]	2001	USA	High	N	N	N	Cross-sectional	System	Bacteriologically confirmed^1,2^
Soltobekova [69]	2022	Kyrgyz Republic	Lower-middle	N	Y	N	Retrospective cohort	System	Bacteriologically confirmed
Story [64]	2020	United Kingdom	High	N	N	N	Cross-sectional	Prison	Bacteriologically confirmed^1,2^
Telisinghe [65]	2014	South Africa	Upper-middle	N	Y	Y	Cross-sectional	Prison	Bacteriologically confirmed^1^
Tsegaye Sahle [66]	2019	Ethiopia	Low	Y	Y	Y	Cross-sectional	Prison	Bacteriologically confirmed
Tulsky [67]	1998	USA	High	N	N	N	Retrospective cohort	Jail	NR
Velen [70]	2021	South Africa	Upper-middle	N	Y	Y	Cross-sectional	Prison	Bacteriologically confirmed
Wang [71]	2023	China	Upper-middle	Y	Y	Y	Cross-sectional	Prison	Bacteriologically confirmed^1^

Legend: Income = national income acc. the World Bank Income Classification [73]; Alg. WHO = screening algorithms acc. [7]; N, no; Y, yes, NR, not reported; PTB, pulmonary tuberculosis, ^1^ = bacteriologically confirmed or clinically diagnosed TB; ^2^ = bacteriologically confirmed TB (only culture positives); ^3^ = bacteriologically confirmed TB (only smear positives); ^4^ = probably active TB (pulmonary lesions compatible with active TB), ^5^ = chest x-rays categorized hierarchically by suggestiveness of infectious TB

Publications with retrospective study designs primarily reported clinical settings managed solely by detention health services, with public health services providing additional support for health personnel and laboratory capacities. In contrast, studies with cross-sectional or prospective designs generally enhanced existing healthcare services in detention facilities through additional study personnel to conduct specific screening procedures.

The reported participant characteristics included TB signs and symptoms, age, sex distribution, and TB history, which often diverged from the average detention population outlined in the review protocol due to differing study objectives.

Fourteen studies were conducted in TB/HIV high-burden countries (HBCs), suggesting a higher prevalence of HIV among incarcerated individuals and a greater likelihood of TB-HIV co-infection [28,42,48–49,51–52,56–57,62,65–66,70–72]. The remaining study populations were not representative due to various issues, e.g., design-introduced selection bias [36,68]. Additionally, variations were noted in studies conducted before 1992, prior to the WHO introduction of the Directly Observed Treatment, Short-Course (DOTS) strategy [38,44,55,60]. Furthermore, some studies excluded participants with former TB disease from enrolment or data analysis [56,58,69,71].

Reported outcomes included prevalence and incidence of TB cases, case-finding rates (CFR), numbers of individuals suspected of having TB identified by various screening methods, results from diagnostic tests, patterns of transmission, results of drug susceptibility tests, and effectiveness of screening and treatment measures. Nonetheless, diagnostic accuracy parameters were typically reported for entire screening programs rather than for entry screening. Many publications described screening approaches that encompassed admission screenings as well as periodic mass and exit screenings, with passive methods for symptomatic residents supplementing some screening approaches.

Regarding the definition of active PTB, one-third of the studies lacked information. Ten studies applied bacteriologically confirmed or clinically diagnosed TB, whereas another added probably active TB (pulmonary lesions compatible with active TB). One investigation relied on CXR, which was categorized hierarchically by the suggestiveness of infectious TB. In contrast, eight studies only accepted bacteriologically confirmed TB. Two other investigations restricted their definition to culture-positive PTB. Another four publications reported restrictions to culture-positivity or smear-positivity but simultaneously broadened their PTB definition to clinically diagnosed cases. As reported in the publications, the definition of active PTB as the target disease was based on established national or WHO criteria of that time.

**Figure 3:**
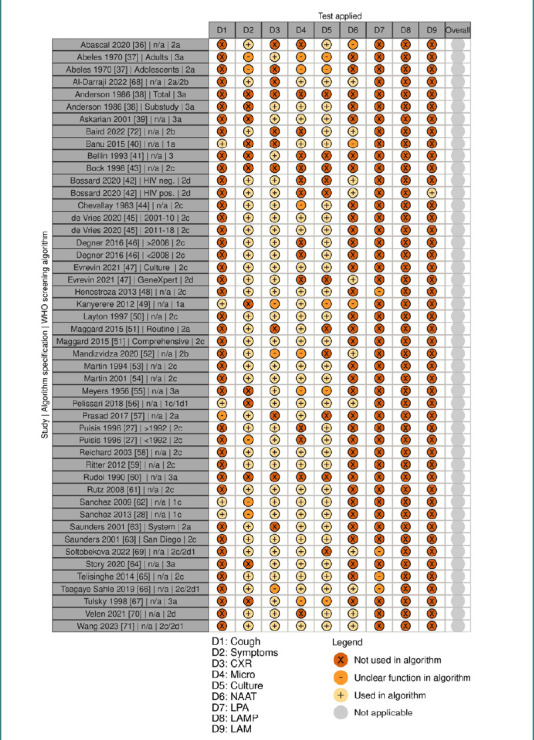
Screening algorithms applied on admission. Legend: CXR, chest X-ray; Micro, microscopy; NAAT, nucleic acid amplification test; LPA, line probe assay; LAMP, loop-mediated isothermal amplification; LAM, lipoarabinomannan test; 1 = both algorithms may apply; The figure was generated with the robvis web app [32].

Because nine of 39 publications reported the application of two different screening algorithms [27,37,38,42,45–47,51,63], the overall score of algorithms encompassed 48 entry screening procedures (Figure 3). The number of new arrivals reported for the 48 algorithms ranged from 34 to 452651 persons. The range of screened persons on admission comprised 34 to 369218 individuals. For 13 of the 48 algorithms, the publications did not provide the total number of newly admitted individuals. Regarding persons screened on admission, absolute numbers were lacking for 13 algorithms. The reporting of individuals suspected of PTB was unavailable for 17 algorithms. Also, data on PTB cases identified in total, the portion of incident cases, cases already under treatment when identified, clinically diagnosed, and bacteriologically confirmed cases were scattered. When data on prevalent and incident TB cases were available, the reporting often condensed the findings from different screening time points or even prevalent and incident cases.

Linking the entry screening procedures to the established WHO screening algorithms [7], 19 procedures were equal to the algorithm ‘2c’, which starts with symptoms screening, followed by CXR, followed by sputum smear microscopy. In summary, 32 algorithms started with TB symptom screening. In contrast, cough screening was performed first in five algorithms. Overall, 37 algorithms included CXR screening, making this technology the most popular among the studies. Nine algorithms relied on CXR as the only screening test. Of these, eight used smear microscopy as a diagnostic test. Three other publications remained imprecisely concerning CXR screening at entry. None applied CXR and GeneXpert alone. However, GeneXpert was once combined with cough and CXR and ten times with TB symptoms screening, six of which also included CXR.

Among the diagnostic tests, lipoarabinomannan testing (TB-LAM) was applied once to individuals living with HIV in Malawi [42]. Sputum culture was the most frequent diagnostic test (*n* = 32), although it was once restricted to specific conditions. The extent of sputum culture used for TB diagnosis was unclear in four publications. Similarly, the application of smear microscopy was not precisely detailed in six publications, though it served as a diagnostic tool in 28 studies. Nucleic acid amplification tests (NAATs), such as GeneXpert, were featured in 11 algorithms, with their role in TB diagnosis insufficiently described in three studies, as was the case for first-line line probe assays (FL LPA) in four studies. No publication reported loop-mediated isothermal amplification (TB-LAMP) as a diagnostic test.

When funding data were available, the funding sources mentioned were mostly government-based, non-governmental foundations, universities, or popular international initiatives focused on TB or HIV, such as the TB REACH Initiative. The study by Evrevin and colleagues was the only one financed by the industry, specifically by Cepheid Industry, the manufacturer of GeneXpert [47].

## DISCUSSION

We aimed to identify the most accurate screening algorithm to detect individuals with active PTB upon detention admission. However, our systematic review found no publications providing the necessary data for the planned meta-analyses. As a result, the current methodology could not determine which TB screening strategy at entry is the most accurate in terms of balanced diagnostic accuracy.

Despite this, our review of publications on TB screening upon admission investigated differences in entry screening applications between detention settings in TB HBCs and other settings with less limited structural, financial, and personnel resources [74]. All studies used established screening and diagnostic tests, following WHO guidelines. Smear microscopy and sputum culture were standard diagnostic tests regardless of income status. However, two HBC algorithms applied smear microscopy exclusively to confirm active PTB. We found other critical differences in cough screening performed in five studies from Bangladesh, Malawi, and Brazil [28,40,49,56,62] and the NAAT application. Despite their higher cost, NAAT techniques were employed in nine HBCs [36,40,42,49,52,56,66,69–72], whereas only one high-income location utilized NAAT [47]. The cause might be that NAAT for TB was introduced around 2010, and only four high-income-country publications were more recent. Nevertheless, having evidence from 22 studies published in 2010 or later represents a potential for future meta-analysis of relevant findings.

The urgency of TB control in detention settings, driven by the high TB burden, necessitates the use of modern diagnostic techniques for effective case finding, underscoring the need for action and funding willingness for TB control programs and their optimization. This need is evident across various detention settings, as highlighted by our analysis [27,28,36–67]. Furthermore, the characteristics of persons upon admission, TB epidemiology, and transmission risks differ significantly from those of persons during residency or upon release from confinement [36,40,48]. The stress associated with admissions can affect individuals’ perceptions and responsiveness to medical examinations, including TB entry screening [44,50,62].

We agree with Henostroza and Pelissari that cough or TB symptom screening alone is insufficient for effective case detection upon admission [48,56]. Investigations in the Berlin detention system in Germany found that 25% of PTB patients were asymptomatic at diagnosis [75]. It is also a question of timing [47,51], given the volatility of new admissions due to short-term detentions or transfers, as demonstrated by the high dropout numbers reported [27,38,41,42,44,49,50,52,59,61,67]. Therefore, enhancing TB control in detention requires rapid, highly sensitive screening and diagnostic tests, alongside other critical components for designing effective TB and HIV screening programs [51].

Given the rapid spread of TB among incarcerated individuals, it is essential to accurately determine TP, FP, TN, and FN values for all new admissions to derive reliable diagnostic accuracy estimates. Relying on TB cases diagnosed weeks after admission to assess the sensitivity of screening methods [43,46] can be misleading, as it underestimates the potential for transmission during the initial stages of residency. Although it is common practice in TB screening programs to conduct diagnostic testing based solely on presumptive cases [8], this approach may not capture the full scope of TB spread. Nonetheless, two Zambian studies included all new arrivals in sputum collection and testing [48,51]. Unfortunately, these studies did not provide the necessary admission data for a thorough analysis.

Our study highlights the significant unmet need for diagnostic accuracy studies focused on TB entry screening. Moreover, publications should clearly delineate the entry screening processes to better calculate case-finding rates and decide on the most effective TB control strategies in detention systems. This is crucial for inferences about the communities from which incarcerated persons originate, especially if governments aim to improve TB case detection and subsequent treatment outcomes in the general population [36]. The lack of diagnostic accuracy studies on entry might be related to the reliance of detention studies on good quality community data, which, from the authorities’ perspective, might negate the need for such studies. This perspective may lead to the diversion of limited resources away from determining which screening algorithms are most effective at diagnosing TB upon entry. This policy approach could potentially limit the scope and impact of findings. Eventually, governments should enable criminal justice systems to perform efficient TB screening on admission, during residency, and exit and offer treatment according to current guidelines.

### Limitations

Our research is limited by the inherent biases of a systematic review methodology, which restricts the ability to generate adequate findings. This limitation also extends to the utilization of non-peer-reviewed records from the grey literature. The design of the studies reviewed in the full text did not include any diagnostic accuracy studies, thus failing to answer our research question. This indicates that diagnostic accuracy-based approaches may not be suitable in the current landscape of TB control in criminal justice systems. Additionally, the methodology did not account for missing data, representing another significant drawback.

Future research should potentially rely on real-world evidence (RWE) studies and conceptualize a research approach that investigates the overall test performance related to the underlying TB prevalence, such as CFR, which is more likely to generate evidence sufficient to rank TB entry screening algorithms according to their accuracy. With their work, Cords and colleagues have already created a basis for an NMA, at least in theory [76]. That should be the subject of future research efforts.

The fact that the methodology of a systematic review is so restrictive in its qualitative requirements may have been more of a hindrance than a benefit to investigating the diagnostic accuracy of TB case-finding measures using the strategy chosen here. Herein, its methodology introduces an additional risk of attrition bias and increases statistical heterogeneity. Nonetheless, our qualitative findings can contribute to further investigations of TB entry screening in custody. Furthermore, the review protocol stated to contact the authors if a study can provide useful data but is not available in the publication. We refrained from that request, as 17 of the 39 studies were published before 2010. Waiting for author feedback may fail even for articles published after 2010 but older than five years.

Although the current approach failed to deliver the evidence requested and all criticisms related to the methodology applied, the failure of the present investigation uncovers the need for data in the realm of TB entry control strategies in detention settings. The evident lack of targeted diagnostic accuracy studies underscores the importance and potential impact of this research. This gap may indeed reflect the significant value and necessity of further investigative efforts in this area.

## CONCLUSION

There is a paucity of data on the diagnostic accuracy of TB screening algorithms for entry into custody across the globe. We recommend that global TB initiatives set up multi-site studies to investigate the diagnostic accuracy of TB screening on admission in low- and high-prevalence criminal justice systems. Furthermore, NMAs of these studies should be conducted to provide policymakers and public health experts with the information needed to develop or refine TB screening protocols in detention environments.

## Personal thanks

We thank Vasumathi Sriganesh for her advice concerning search strategy development. We also thank Ashish Verma (AV) for his assistance with the literature screening procedures.
