# Supplementary material for: Diagnostic accuracy of active pulmonary tuberculosis screening during detention admission: a systematic review
Source: J Med Life. 2024 Jul;17(7):671–81. doi: 10.25122/jml-2024-0155 (PMC11493172; doi:10.25122/jml-2024-0155)
Supplement: Supplementary file 1 [file JMedLife-17-671-s001.pdf]

1. PUBMED SEARCH STRATEGY

Date Run: 09/03/2022

Search number	Query	Sort By	Filters	Results
7	((“Prisons”[Mesh] OR “Prisoners”[Mesh] OR prison*[tw] OR penal[tw] OR jail*[tw] OR reformator*[tw] OR custodial[tw] OR custody[tw] OR gaol*[tw] OR remand*[tw] OR penitentiar*[tw] OR detention*[tw] OR correctional[tw] OR detainee*[tw] OR inmate*[tw] OR imprison*[tw] OR confinement[tw] OR incarcerat*[tw] OR cellmate*[tw] OR lockup*[tw] OR penitentiar*[tw] OR penal institution*[tw] OR detention facilit*[tw] OR bastille*[tw] OR clink*[tw] OR dungeon*[tw] OR guardhouse*[tw] OR slammer*[tw] OR stockade*[tw] OR statesville*[tw] OR up the river[tw] OR brig*[tw] OR bullpen*[tw] OR jailhouse*[tw] OR rack*[tw] OR solitar*[tw] OR stir*[tw] OR “black hole*”[tw] OR “detention camp*”[tw] OR “house of correction”[tw]) AND (“Mass Screening”[Mesh] OR “Mandatory Testing”[Mesh] OR screen*[tw] OR “case finding”[tw] OR “case-finding”[tw] OR casefinding[tw] OR “cases finding”[tw] OR “case identification”[tw] OR “cases identification”[tw] OR testing[tw] OR “rapid test”[tw] OR “rapid tests”[tw] OR “Early diagnosis”[Mesh] OR early diagnos*[tw] OR early detect*[tw] OR early test*[tw] OR “clinical evaluation”[tw] OR “clinical evaluations”[tw])) AND (((“Mass Screening”[Mesh] OR “Mandatory Testing”[Mesh] OR screen*[tw] OR “case finding”[tw] OR “case-finding”[tw] OR casefinding[tw] OR “cases finding”[tw] OR “case identification”[tw] OR “cases identification”[tw] OR testing[tw] OR “rapid test”[tw] OR “rapid tests”[tw] OR “Early diagnosis”[Mesh] OR early diagnos*[tw] OR early detect*[tw] OR early test*[tw] OR “clinical evaluation”[tw] OR “clinical evaluations”[tw]) AND (“Tuberculosis”[Mesh] OR “Mycobacterium tuberculosis”[Mesh] OR “Mycobacterium avium”[Mesh] OR “Mycobacterium bovis”[Mesh] OR tuberc*[tw] OR “Kochs Disease”[tw] OR “Koch’s Disease”[tw] OR “Koch Disease”[tw] OR TB[tw] OR LTB[tw] OR LTBI[tw] OR DRTB[tw] OR “DR-TB”[tw] OR XDRTB[tw] OR “XDR-TB”[tw] OR MDRTB[tw] OR “MDR-TB”[tw] OR “Mycobacterium bovis”[tw] OR “M. bovis”[tw] OR “Mycobacterium avium”[tw] OR “M. avium”[tw])) OR (Tuberculosis/diagnosis[Mesh]))			667
6	((“Mass Screening”[Mesh] OR “Mandatory Testing”[Mesh] OR screen*[tw] OR “case finding”[tw] OR “case-finding”[tw] OR casefinding[tw] OR “cases finding”[tw] OR “case identification”[tw] OR “cases identification”[tw] OR testing[tw] OR “rapid test”[tw] OR “rapid tests”[tw] OR “Early diagnosis”[Mesh] OR early diagnos*[tw] OR early detect*[tw] OR early test*[tw] OR “clinical evaluation”[tw] OR “clinical evaluations”[tw]) AND (“Tuberculosis”[Mesh] OR “Mycobacterium tuberculosis”[Mesh] OR “Mycobacterium avium”[Mesh] OR “Mycobacterium bovis”[Mesh] OR tuberc*[tw] OR “Kochs Disease”[tw] OR “Koch’s Disease”[tw] OR “Koch Disease”[tw] OR TB[tw] OR LTB[tw] OR LTBI[tw] OR DRTB[tw] OR “DR-TB”[tw] OR XDRTB[tw] OR “XDR-TB”[tw] OR MDRTB[tw] OR “MDR-TB”[tw] OR “Mycobacterium bovis”[tw] OR “M. bovis”[tw] OR “Mycobacterium avium”[tw] OR “M. avium”[tw])) OR (Tuberculosis/diagnosis[Mesh])			74061
5	Tuberculosis/diagnosis[Mesh]			52742
4	(“Mass Screening”[Mesh] OR “Mandatory Testing”[Mesh] OR screen*[tw] OR “case finding”[tw] OR “case-finding”[tw] OR casefinding[tw] OR “cases finding”[tw] OR “case identification”[tw] OR “cases identification”[tw] OR testing[tw] OR “rapid test”[tw] OR “rapid tests”[tw] OR “Early diagnosis”[Mesh] OR early diagnos*[tw] OR early detect*[tw] OR early test*[tw] OR “clinical evaluation”[tw] OR “clinical evaluations”[tw]) AND (“Tuberculosis”[Mesh] OR “Mycobacterium tuberculosis”[Mesh] OR “Mycobacterium avium”[Mesh] OR “Mycobacterium bovis”[Mesh] OR tuberc*[tw] OR “Kochs Disease”[tw] OR “Koch’s Disease”[tw] OR “Koch Disease”[tw] OR TB[tw] OR LTB[tw] OR LTBI[tw] OR DRTB[tw] OR “DR-TB”[tw] OR XDRTB[tw] OR “XDR-TB”[tw] OR MDRTB[tw] OR “MDR-TB”[tw] OR “Mycobacterium bovis”[tw] OR “M. bovis”[tw] OR “Mycobacterium avium”[tw] OR “M. avium”[tw])			31165
3	“Tuberculosis”[Mesh] OR “Mycobacterium tuberculosis”[Mesh] OR “Mycobacterium avium”[Mesh] OR “Mycobacterium bovis”[Mesh] OR tuberc*[tw] OR “Kochs Disease”[tw] OR “Koch’s Disease”[tw] OR “Koch Disease”[tw] OR TB[tw] OR LTB[tw] OR LTBI[tw] OR DRTB[tw] OR “DR-TB”[tw] OR XDRTB[tw] OR “XDR-TB”[tw] OR MDRTB[tw] OR “MDR-TB”[tw] OR “Mycobacterium bovis”[tw] OR “M. bovis”[tw] OR “Mycobacterium avium”[tw] OR “M. avium”[tw]			332645
2	“Mass Screening”[Mesh] OR “Mandatory Testing”[Mesh] OR screen*[tw] OR “case finding”[tw] OR “case-finding”[tw] OR casefinding[tw] OR “cases finding”[tw] OR “case identification”[tw] OR “cases identification”[tw] OR testing[tw] OR “rapid test”[tw] OR “rapid tests”[tw] OR “Early diagnosis”[Mesh] OR early diagnos*[tw] OR early detect*[tw] OR early test*[tw] OR “clinical evaluation”[tw] OR “clinical evaluations”[tw]			1820292
1	“Prisons”[Mesh] OR “Prisoners”[Mesh] OR prison*[tw] OR penal[tw] OR jail*[tw] OR reformator*[tw] OR custodial[tw] OR custody[tw] OR gaol*[tw] OR remand*[tw] OR penitentiar*[tw] OR detention*[tw] OR correctional[tw] OR detainee*[tw] OR inmate*[tw] OR imprison*[tw] OR confinement[tw] OR incarcerat*[tw] OR cellmate*[tw] OR lockup*[tw] OR penitentiar*[tw] OR penal institution*[tw] OR detention facilit*[tw] OR bastille*[tw] OR clink*[tw] OR dungeon*[tw] OR guardhouse*[tw] OR slammer*[tw] OR stockade*[tw] OR statesville*[tw] OR up the river[tw] OR brig*[tw] OR bullpen*[tw] OR jailhouse*[tw] OR rack*[tw] OR solitar*[tw] OR stir*[tw] OR “black hole*”[tw] OR “detention camp*”[tw] OR “house of correction”[tw]			216347

© 2024 by the authors. This article is an open access article distributed under the terms and conditions of the Creative Commons Attribution (CC BY 4.0) license.

2. COCHRANE SEARCH STRATEGY

Date Run: 09/03/2022

ID	Search	Hits
#1	MeSH descriptor: [Prisoners] explode all trees	344
#2	MeSH descriptor: [Prisoners] explode all trees	344
#3	(prison*):ti,ab,kw OR (penal*):ti,ab,kw OR (jail*):ti,ab,kw OR (reformator*):ti,ab,kw OR (custodial):ti,ab,kw (Word variations have been searched)	2158
#4	(custody):ti,ab,kw OR (gaol*):ti,ab,kw OR (remand*):ti,ab,kw OR (penitentiar*):ti,ab,kw OR (detention*):ti,ab,kw (Word variations have been searched)	421
#5	(correctional):ti,ab,kw OR (detainee*):ti,ab,kw OR (inmate*):ti,ab,kw OR (imprison*):ti,ab,kw OR (confinement):ti,ab,kw (Word variations have been searched)	51907
#6	(incarcerat*):ti,ab,kw OR (cellmate*):ti,ab,kw OR (lockup*):ti,ab,kw OR (penal institution*):ti,ab,kw OR (detention facilit*):ti,ab,kw (Word variations have been searched)	865
#7	(bastille*):ti,ab,kw OR (clink*):ti,ab,kw OR (dungeon*):ti,ab,kw OR (guardhouse*):ti,ab,kw OR (slammer*):ti,ab,kw (Word variations have been searched)	2
#8	(stockade*):ti,ab,kw OR (statesville*):ti,ab,kw OR (up the river):ti,ab,kw OR (brig*):ti,ab,kw OR (bullpen*):ti,ab,kw (Word variations have been searched)	2676
#9	(jailhouse*):ti,ab,kw OR (rack*):ti,ab,kw OR (solitar*):ti,ab,kw OR (stir*):ti,ab,kw OR (black hole*):ti,ab,kw (Word variations have been searched)	1889
#10	(detention camp*):ti,ab,kw OR (house of correction):ti,ab,kw OR (houses of correction):ti,ab,kw (Word variations have been searched)	221
#11	{OR #1-#10}	58330
#12	MeSH descriptor: [Mass Screening] explode all trees	4058
#13	MeSH descriptor: [Mandatory Testing] explode all trees	3
#14	(screen*):ti,ab,kw OR (case finding):ti,ab,kw OR (case-finding):ti,ab,kw OR (casefinding):ti,ab,kw OR (cases finding):ti,ab,kw (Word variations have been searched)	100057
#15	(case identification):ti,ab,kw OR (cases identification):ti,ab,kw OR (testing):ti,ab,kw OR (rapid test):ti,ab,kw OR (rapid tests):ti,ab,kw (Word variations have been searched)	411736
#16	MeSH descriptor: [Early Diagnosis] explode all trees	1975
#17	(early diagnos*):ti,ab,kw OR (early detect*):ti,ab,kw OR (early test*):ti,ab,kw OR (clinical evaluation):ti,ab,kw OR (clinical evaluations):ti,ab,kw (Word variations have been searched)	382297
#18	{OR #12-#17}	710870
#19	MeSH descriptor: [Tuberculosis] explode all trees	2459
#20	MeSH descriptor: [Mycobacterium tuberculosis] explode all trees	348
#21	MeSH descriptor: [Mycobacterium avium] explode all trees	11
#22	MeSH descriptor: [Mycobacterium bovis] explode all trees	87
#23	(tuberc*):ti,ab,kw OR (Kochs Disease):ti,ab,kw OR (Koch's Disease):ti,ab,kw OR (Koch Disease):ti,ab,kw OR (TB):ti,ab,kw (Word variations have been searched)	9332
#24	(LTB):ti,ab,kw OR (LTBI):ti,ab,kw OR (DRTB):ti,ab,kw OR (DR-TB):ti,ab,kw OR (XDRTB):ti,ab,kw (Word variations have been searched)	338
#25	(XDR-TB):ti,ab,kw OR (MDRTB):ti,ab,kw OR (MDR-TB):ti,ab,kw OR (Mycobacterium bovis):ti,ab,kw OR (M. bovis):ti,ab,kw (Word variations have been searched)	518
#26	(Mycobacterium avium):ti,ab,kw OR (M. avium):ti,ab,kw (Word variations have been searched)	303
#27	{OR #19-#26}	9866
#28	{AND #18, #27}	4969
#29	MeSH descriptor: [Tuberculosis] explode all trees	2459
#30	MeSH descriptor: [Diagnosis] explode all trees	348517
#31	{AND #29, #30}	1007
#32	{OR #28, #31}	5358
#33	{AND #11, #18, #32}	228

© 2024 by the authors. This article is an open access article distributed under the terms and conditions of the Creative Commons Attribution (CC BY 4.0) license.

3. GLOBAL INDEX MEDICUS

Same search strategy as for Pubmed. Date Run: 09/03/2022

ID	Search	Hits
FINAL STRATEGY<br><br>#1 AND #2 AND #6	tw:((tw:(tw:((mh:(prisons)) OR (mh:(prisoners)) OR (tw:(prison*)) OR (tw:(penal)) OR (tw:(jail*)) OR (tw:(reformator*)) OR (tw:(custodial)) OR (tw:(custody)) OR (tw:(gaol*)) OR (tw:(remand*)) OR (tw:(penitentiar*)) OR (tw:(detention*)) OR (tw:(correctional)) OR (tw:(detainee*)) OR (tw:(inmate*)) OR (tw:(imprison*)) OR (tw:(confinement)) OR (tw:(incarcerat*)) OR (tw:(cellmate*)) OR (tw:(lockup*)) OR (tw:(penal institution*)) OR (tw:(detention facilit*)) OR (tw:(bastille*)) OR (tw:(clink*)) OR (tw:(dungeon*)) OR (tw:(guardhouse*)) OR (tw:(slammer*)) OR (tw:(stockade*)) OR (tw:(statesville*)) OR (tw:(up the river)) OR (tw:(brig*)) OR (tw:(bullpen*)) OR (tw:(jailhouse*)) OR (tw:(rack*)) OR (tw:(solitar*)) OR (tw:(stir*)) OR (tw:(black hole*)) OR (tw:(detention camp*)) OR (tw:(house of correction)) OR (tw:(houses of correction))))) AND (tw:(tw:((mh:(mass screening)) OR (mh:(mandatory testing)) OR (tw:(screen*)) OR (tw:("case finding")) OR (tw:("case-finding")) OR (tw:(casefinding)) OR (tw:("cases finding")) OR (tw:("case identification")) OR (tw:("cases identification")) OR (tw:(testing)) OR (tw:("rapid test")) OR (tw:("rapid tests")) OR (mh:("early diagnosis")) OR (tw:(early diagnos*)) OR (tw:(early detect*)) OR (tw:(early test*)) OR (tw:("clinical evaluation")) OR (tw:("clinical evaluations"))))) AND (tw:(tw:((tw:(tw:((tw:(w:((mh:(mass screening)) OR (mh:(mandatory testing)) OR (tw:(screen*)) OR (tw:("case finding")) OR (tw:("case-finding")) OR (tw:(casefinding)) OR (tw:("cases finding")) OR (tw:("case identification")) OR (tw:("cases identification")) OR (tw:(testing)) OR (tw:("rapid test")) OR (tw:("rapid tests")) OR (mh:("early diagnosis")) OR (tw:(early diagnos*)) OR (tw:(early detect*)) OR (tw:(early test*)) OR (tw:("clinical evaluation")) OR (tw:("clinical evaluations"))))) AND (tw:(tw:((mh:(tuberculosis)) OR (mh:("Mycobacterium tuberculosis")) OR (mh:("Mycobacterium avium")) OR (mh:("Mycobacterium bovis")) OR (tw:(tuberc*)) OR (tw:("Kochs Disease")) OR (tw:("Koch's Disease")) OR (tw:("Koch Disease")) OR (tw:(tb)) OR (tw:(ltb)) OR (tw:(ltbi)) OR (tw:(drtb)) OR (tw:("dr-tb")) OR (tw:(xdrtb)) OR (tw:("xdr-tb")) OR (tw:(mdrtb)) OR (tw:("mdr-tb")) OR (tw:("Mycobacterium bovis")) OR (tw:("m. bovis")) OR (tw:("Mycobacterium avium")) OR (tw:("m. avium")))))))) OR (tw:(tw:((mh:(tuberculosis)) AND (mh:(diagnosis))))))))	162
#6 MERGE #4 OR #5	tw:((tw:(tw:((tw:(w:((mh:(mass screening)) OR (mh:(mandatory testing)) OR (tw:(screen*)) OR (tw:("case finding")) OR (tw:("case-finding")) OR (tw:(casefinding)) OR (tw:("cases finding")) OR (tw:("case identification")) OR (tw:("cases identification")) OR (tw:(testing)) OR (tw:("rapid test")) OR (tw:("rapid tests")) OR (mh:("early diagnosis")) OR (tw:(early diagnos*)) OR (tw:(early detect*)) OR (tw:(early test*)) OR (tw:("clinical evaluation")) OR (tw:("clinical evaluations"))))) AND (tw:(tw:((mh:(tuberculosis)) OR (mh:("Mycobacterium tuberculosis")) OR (mh:("Mycobacterium avium")) OR (mh:("Mycobacterium bovis")) OR (tw:(tuberc*)) OR (tw:("Kochs Disease")) OR (tw:("Koch's Disease")) OR (tw:("Koch Disease")) OR (tw:(tb)) OR (tw:(ltb)) OR (tw:(ltbi)) OR (tw:(drtb)) OR (tw:("dr-tb")) OR (tw:(xdrtb)) OR (tw:("xdr-tb")) OR (tw:(mdrtb)) OR (tw:("mdr-tb")) OR (tw:("Mycobacterium bovis")) OR (tw:("m. bovis")) OR (tw:("Mycobacterium avium")) OR (tw:("m. avium")))))))) OR (tw:(tw:((mh:(tuberculosis)) AND (mh:(diagnosis))))))	
#5 ADD	tw:((mh:(tuberculosis)) AND (mh:(diagnosis)))	
#4 MERGE #2 AND #3	tw:((tw:(w:((mh:(mass screening)) OR (mh:(mandatory testing)) OR (tw:(screen*)) OR (tw:("case finding")) OR (tw:("case-finding")) OR (tw:(casefinding)) OR (tw:("cases finding")) OR (tw:("case identification")) OR (tw:("cases identification")) OR (tw:(testing)) OR (tw:("rapid test")) OR (tw:("rapid tests")) OR (mh:("early diagnosis")) OR (tw:(early diagnos*)) OR (tw:(early detect*)) OR (tw:(early test*)) OR (tw:("clinical evaluation")) OR (tw:("clinical evaluations"))))) AND (tw:(tw:((mh:(tuberculosis)) OR (mh:("Mycobacterium tuberculosis")) OR (mh:("Mycobacterium avium")) OR (mh:("Mycobacterium bovis")) OR (tw:(tuberc*)) OR (tw:("Kochs Disease")) OR (tw:("Koch's Disease")) OR (tw:("Koch Disease")) OR (tw:(tb)) OR (tw:(ltb)) OR (tw:(ltbi)) OR (tw:(drtb)) OR (tw:("dr-tb")) OR (tw:(xdrtb)) OR (tw:("xdr-tb")) OR (tw:(mdrtb)) OR (tw:("mdr-tb")) OR (tw:("Mycobacterium bovis")) OR (tw:("m. bovis")) OR (tw:("Mycobacterium avium")) OR (tw:("m. Avium"))))))	
#3 TB	tw:((mh:(tuberculosis)) OR (mh:("Mycobacterium tuberculosis")) OR (mh:("Mycobacterium avium")) OR (mh:("Mycobacterium bovis")) OR (tw:(tuberc*)) OR (tw:("Kochs Disease")) OR (tw:("Koch's Disease")) OR (tw:("Koch Disease")) OR (tw:(tb)) OR (tw:(ltb)) OR (tw:(ltbi)) OR (tw:(drtb)) OR (tw:("dr-tb")) OR (tw:(xdrtb)) OR (tw:("xdr-tb")) OR (tw:(mdrtb)) OR (tw:("mdr-tb")) OR (tw:("Mycobacterium bovis")) OR (tw:("m. bovis")) OR (tw:("Mycobacterium avium")) OR (tw:("m. avium")))	
#2 Active case finding	tw:((mh:(mass screening)) OR (mh:(mandatory testing)) OR (tw:(screen*)) OR (tw:("case finding")) OR (tw:("case-finding")) OR (tw:(casefinding)) OR (tw:("cases finding")) OR (tw:("case identification")) OR (tw:("cases identification")) OR (tw:(testing)) OR (tw:("rapid test")) OR (tw:("rapid tests")) OR (mh:("early diagnosis")) OR (tw:(early diagnos*)) OR (tw:(early detect*)) OR (tw:(early test*)) OR (tw:("clinical evaluation")) OR (tw:("clinical evaluations")))	
#1 Prison settings	tw:((mh:(prisons)) OR (mh:(prisoners)) OR (tw:(prison*)) OR (tw:(penal)) OR (tw:(jail*)) OR (tw:(reformator*)) OR (tw:(custodial)) OR (tw:(custody)) OR (tw:(gaol*)) OR (tw:(remand*)) OR (tw:(penitentiar*)) OR (tw:(detention*)) OR (tw:(correctional)) OR (tw:(detainee*)) OR (tw:(inmate*)) OR (tw:(imprison*)) OR (tw:(confinement)) OR (tw:(incarcerat*)) OR (tw:(cellmate*)) OR (tw:(lockup*)) OR (tw:(penal institution*)) OR (tw:(detention facilit*)) OR (tw:(bastille*)) OR (tw:(clink*)) OR (tw:(dungeon*)) OR (tw:(guardhouse*)) OR (tw:(slammer*)) OR (tw:(stockade*)) OR (tw:(statesville*)) OR (tw:(up the river)) OR (tw:(brig*)) OR (tw:(bullpen*)) OR (tw:(jailhouse*)) OR (tw:(rack*)) OR (tw:(solitar*)) OR (tw:(stir*)) OR (tw:(black hole*)) OR (tw:(detention camp*)) OR (tw:(house of correction)) OR (tw:(houses of correction)))	

© 2024 by the authors. This article is an open access article distributed under the terms and conditions of the Creative Commons Attribution (CC BY 4.0) license.

4. GREY LITERATURE SEARCH - WEBSITES

As practiced by the ECDC working group, the grey literature search was conducted by visiting the websites listed below (European Centre for Disease Prevention and Control, 6–7, Appendix 1).

Conference abstract websites

- International Union for Tuberculosis and Lung Disease (http://www.theunion.org/)
- European Respiratory Society (http://www.ersnet.org/)
- American Respiratory Society (https://www.thoracic.org/)
- International Corrections and Prisons Association (ICPA, http://icpa.ca/)
- American Correctional Association (http://www.aca.org/aca_prod_imis/aca_member)
- Experiencing Prison 7th Global Conference (http://www.inter-disciplinary.net/probing-the-boundaries/persons/experiencing-prison/)
- National Conference on Correctional Health Care (http://www.ncchc.org/national-conference)

Other websites

1. Guidelines
- Guidelines International Network (http://www.g-i-n.net/)
- NICE guidelines (https://www.evidence.nhs.uk/)

2. Organizations and Institutes
- WHO – Health in Prisons Programme (HIPP) (http://www.euro.who.int/prisons)
- WHO – EU (http://www.euro.who.int/en/home)
- WHO – IRIS (http://apps.who.int/iris/)
- Council of Europe/POMPIDOU Group (http://www.coe.int/T/DG3/Pompidou/
- AboutUs/default_en.asp), and other Council of Europe documents
- UNODC (http://www.unodc.org/)
- ECDC (http://ecdc.europa.eu/en/Pages/home.aspx)
- Public Health England (PHE) (http://www.gov.uk)
- European Monitoring Centre for Drugs and Drug Addiction (EMCDDA) (http://www.emcdda.europa.eu/)
- International Corrections and Prisons Association (ICPA, http://icpa.ca/)

3. Bibliographies
- Campbell Collaboration (http://www.campbellcollaboration.org/)
- Bibliography on HIV/AIDS and Hepatitis C in prisons (http://www.aidslaw.ca/)
- IDEAS (https://ideas.repec.org/)
- Evidence in Health and Social Care (NHS Evidence, https://www.evidence.nhs.uk/)
- Open grey (http://www.opengrey.eu)

© 2024 by the authors. This article is an open access article distributed under the terms and conditions of the Creative Commons Attribution (CC BY 4.0) license.
